# The Use of Social Media by State Tobacco Control Programs to Promote Smoking Cessation: A Cross-Sectional Study

**DOI:** 10.2196/jmir.3430

**Published:** 2014-07-10

**Authors:** Jennifer C Duke, Heather Hansen, Annice E Kim, Laurel Curry, Jane Allen

**Affiliations:** ^1^RTI InternationalPublic Health Policy ResearchResearch Triangle Park, NCUnited States

**Keywords:** social media, tobacco, smoking, public health, mass media

## Abstract

**Background:**

The promotion of evidence-based cessation services through social media sites may increase their utilization by smokers. Data on social media adoption and use within tobacco control programs (TCPs) have not been reported.

**Objective:**

This study examines TCP use of and activity levels on social media, the reach of TCP sites, and the level of engagement with the content on sites.

**Methods:**

A cross-sectional descriptive study of state TCP social media sites and their content was conducted.

**Results:**

In 2013, 60% (30/50) of TCPs were using social media. Approximately one-quarter (26%, 13/50) of all TCPs used 3 or more social media sites, 24% (12/50) used 2, and 10% (5/50) used 1 site. Overall, 60% (30/50) had a Facebook page, 36% (18/50) had a Twitter page, and 40% (20/50) had a YouTube channel. The reach of social media was different across each site and varied widely by state. Among TCPs with a Facebook page, 73% (22/30) had less than 100 likes per 100,000 adults in the state, and 13% (4/30) had more than 400 likes per 100,000 adults. Among TCPs with a Twitter page, 61% (11/18) had less than 10 followers per 100,000 adults, and just 1 state had more than 100 followers per 100,000 adults. Seven states (23%, 7/30) updated their social media sites daily. The most frequent social media activities focused on the dissemination of information rather than interaction with site users. Social media resources from a national cessation media campaign were promoted infrequently.

**Conclusions:**

The current reach of state TCP social media sites is low and most TCPs are not promoting existing cessation services or capitalizing on social media’s interactive potential. TCPs should create an online environment that increases participation and 2-way communication with smokers to promote free cessation services.

## Introduction

Approximately 18% of US adults are cigarette smokers [1]. Evidence from the National Health Interview Survey indicates that, in 2010, 52% of adult smokers in the United States tried to quit in the past year, but only 6% succeeded [2]. The low success rate for smokers’ quit attempts may be due, in part, to the low proportion (31%) of smokers who used evidence-based interventions as part of their quit attempt [2]. All US state tobacco control programs (TCPs) currently offer free evidence-based smoking cessation services through telephone quitlines, and half also offer Web-based interventions. However, the paid promotion of cessation services is limited because of low levels of state TCP funding [3]. Recent national promotion of cessation services has sporadically increased cessation service utilization for brief time periods since 2012 [4]. Given that the decline in the prevalence of smoking has slowed in recent years [5], sustained and innovative approaches are needed to increase the promotion, utilization, and reach of these interventions to maximize their effectiveness [6-8].

The promotion of evidence-based cessation services through social media sites may be a low-cost strategy to increase their utilization by smokers. Almost 3 in 4 US adults aged 18 years or older use at least 1 social media site [9]. Recent data indicate that 71% of online adults use Facebook and 63% of Facebook users visit the site daily [9]. In 2013, 18% of online adults used Twitter and 8% used Twitter daily [9]. Facebook is widely used by online adults across all age groups (84% of adults aged 18 to 29, 79% of adults aged 30 to 49, 60% of adults aged 50 to 64, and 45% of adults aged 65 or older), whereas online adults using Twitter tend to skew to a younger demographic (31% of adults aged 18 to 29, 19% of adults aged 30 to 49, 9% of adults aged 50 to 64, and 5% of adults aged 65 or older) [9]. Photo-sharing sites are also increasing in popularity; for example, 21% of US adults currently use Pinterest and 17% use Instagram [9]. In addition to linking smokers to existing cessation services, social media sites provide a venue for relevant, credible tobacco cessation messages to reach smokers and their social circles. Studies show that people are using social media to seek health information, share their health experiences, and provide and receive psychological support [10-12]. Social media sites also expand social networks, which may influence behavioral and emotional change [13-15]. Media messages promoting free smoking cessation interventions may be shared among smokers, their families, and their friends, motivating quit attempts.

In keeping with the growth of social media activities in the United States, many states currently use social media sites to disseminate health information [16,17]. The most recent descriptive study found that 82% (41/50) of all state public health departments use Twitter and 56% (28/50) use Facebook [18]. A second study of state health departments found that the reach of health department sites relative to the states’ populations was low and, in most cases, the sites failed to take advantage of the fundamental advantage of this medium: engaging users in interactive communication [19]. Harris and colleagues [20] found that state health departments with social media sites were located in more populated states and had higher per capita health department expenditures compared to those without social media sites.

Recently, state TCPs gained new access to free cessation-related content and promotions of cessation services specifically designed to be shared through social media sites. In 2013, the Centers for Disease Control and Prevention (CDC) aired the second wave of the first federally funded tobacco education campaign, Tips From Former Smokers (*Tips*), which encourages quitting through advertisements that show personal testimonials from ex-smokers battling life-altering smoking-related illnesses. The campaign included a large social media presence; CDC shared *Tips*-related materials and social media links publicly on its website and encouraged the public and practitioners to share its social media content with their networks. CDC’s Facebook and Twitter accounts posted frequently during the campaign, sharing updates and campaign-related information, videos, and images.

This study provides a descriptive overview of state TCP social media sites and the extent to which they are used to disseminate cessation messages and promote quitting services. The purpose of this study is to document the use of state TCP social media sites during 2013, including Facebook, Twitter, YouTube, and others. The extent to which TCPs actively use their social media sites, the reach of TCP sites, and audience interaction and engagement with TCP social media sites are examined. This study also explores the content of messages posted on social media by TCPs, particularly state TCPs’ use of existing social media material from the national *Tips* campaign during the months it aired in 2013.

## Methods

### Sample and Data Collection

To identify state TCPs engaged with social media sites, we conducted an online Google search of each state’s name and the keywords “tobacco program” or “quitline.” We then reviewed the websites for hyperlinks or information on related social media sites, including Facebook, Twitter, YouTube, Pinterest, Instagram, Google+, blogs, and virtual communities. Virtual communities are social networks of individuals who interact online; TCP-sponsored communities that encourage smokers to support one another during quit attempts are included in this study. If no links existed, the quitline service name (eg, Quit Now Kentucky) was used in a secondary Google search to identify social media sites. We also included any tobacco-related social media sites sponsored by a state health department but not obviously affiliated with the TCP or quitline name (eg, Make Smoking History, KanQuit!). The search identified 30 states with Facebook sites (ie, pages), 18 states with Twitter sites (ie, accounts identified by name or “handle”), and 20 YouTube sites (ie, channels) as of December 31, 2013 (Multimedia Appendix 1). Two states had more than 1 social media site on the same platform (eg, 2 Facebook pages), in which case the site focused on tobacco cessation was selected for analysis. Publicly available activity data and metrics on all social media sites were collected using Snagit screen capture software (TechSmith Corporation, Okemose, MI, USA). In addition, the social media monitoring tool radian6 (Salesforce Marketing Cloud, New York, NY, USA) was used to capture posted content by users on Twitter.

### Content Coding

The content of state TCP posts to social media sites was collected and analyzed for the subset of months in 2013 during which the national *Tips* smoking cessation campaign aired (March to June 2013). Using an inductive approach, coders observed a subset of messages to develop the coding scheme [21]. Sites with no TCP activity during this time frame were excluded from the content analysis, for a total of 25 Facebook pages, 16 Twitter accounts, and 16 YouTube channels available for coding. Three researchers independently coded 1123 Facebook posts, 1776 Twitter tweets, and 591 YouTube videos (eg, number of likes, shares, comments, message content). Content coding of the most popular social media sites confirmed their primary communication focus: 70.5% (1252/1776) of the content on Twitter and 67.3% (756/1123) of the content on Facebook was about tobacco cessation (and other topics included secondhand smoke and tobacco control policies). Interrater reliability among coders on 5% of data was 95.7% or higher for all coding categories (kappa=.72).

### Measures

#### Overview

Measurement techniques used to guide social media analysis and management that have been pioneered in the for-profit sector [22] may be applied to the study of state TCP social media sites. Key aspects of social media evaluation include exposure (reach or the number of people reached with a message), engagement (number of people who take action in response to a message), and influence (whether engagement is positive, neutral, or negative in sentiment) [23,24]. This study assessed 2 of these 3 evaluation components: exposure to and engagement with state TCP social media sites. Study measures described herein include the presence of social media sites, TCP site activity, audience reach, audience engagement, and social media site promotional activities.

#### Tobacco Control Program Presence on Social Media

We noted whether TCPs had a presence on social media sites (eg, Facebook, Twitter, YouTube) as of December 31, 2013, and the date of site establishment.

#### Tobacco Control Program Social Media Activity

Site activity was defined as the volume of content posted by the TCP on each social media site (ie, the number of posts, tweets, videos) from January to December 2013.

#### Audience Reach

Audience reach on TCP social media sites was measured by Facebook page likes, Twitter followers, and YouTube views (which may represent more than 1 person, but no individual metric is available) as of December 31, 2013. For example, on Facebook, users may “like” a TCP page, enabling posts by the TCP to be sent to users’ newsfeeds where they are the most likely to be seen. Reach metrics are presented as a total number and also as a proportion of the state adult population. State adult population was calculated using 2010 census adult (aged 18 or older) population data and is reported as reach per 100,000 adults.

#### Audience Engagement

Audience interaction and exchange of messages were assessed across social media sites from March to June 2013. As indicators of action on the part of the audience, they serve as a proxy for engagement with the audience. User activity generates content that may be viewed by other users, thus expanding the reach of the site’s messages. To measure audience engagement, data on the amount and relative proportion of the following variables were collected: (1) individual post likes, comments, and shares on Facebook; (2) retweets, defined as the number of times a TCP post (ie, tweet) was shared by another Twitter user; and (3) video likes, shares, and comments on YouTube [19,23].

#### Promotion of State Cessation Services and the Tips Campaign

Coders documented the presence of a link/URL to the states’ cessation resources (ie, the state quitline phone number or website) in each post on Facebook or Twitter from March to June 2013. All posts during this time frame were also coded for the presence of CDC *Tips* campaign content. This included links to *Tips* campaign materials (eg, television advertisements), online campaign stories of the smokers in the *Tips* ads, and shares of CDC’s posts or tweets about *Tips*. YouTube videos were not coded because they were primarily promoted or linked to Facebook and/or Twitter, where the information was captured and coded.

#### Photo and Video Content

Because research has shown that content with images and video attracts more views [25,26], the presence of either photos or videos for each Facebook or Twitter post from March to June 2013 was coded.

### Analysis

We report the results of the collected metrics and content analysis using simple descriptive statistics, including measures of central tendency, frequencies, and counts in summary charts. Because of the limited presence on many social media sites examined in the study, most measures are reported for the 3 most popular sites: Facebook, Twitter, and YouTube.

## Results

### Presence of Social Media Sites

Of all TCPs, 60% (30/50) used social media in 2013. Nearly one-quarter (26%, 13/50) of all TCPs used 3 or more social media sites, 24% (12/50) used 2 sites, and 10% (5/50) used 1 site. The use of Facebook was most common, with 60% (30/50) of state TCPs using the site. Twitter was used by 36% (18/50) of state TCPs and 40% (20/50) had a YouTube channel. In addition to Facebook, Twitter, and YouTube, few state TCPs offered other social media sites. Three states used Pinterest (Louisiana, Mississippi, and Oregon), 2 states had their own blog (California and New Hampshire), and 1 state offered Google+ (New York). In addition, 3 states provided a virtual community site for smoking cessation (Arizona, Connecticut, and New York). No states used Instagram in 2013.

### Tobacco Control Program Site Activity

**Figure 1:**
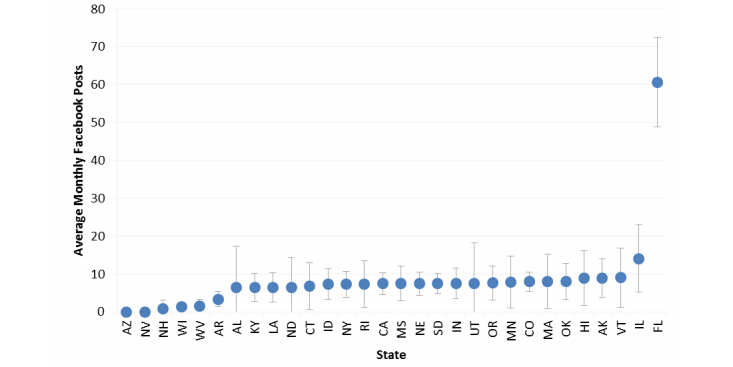
Average number of monthly Facebook posts by state tobacco control programs (n=30), January to December 2013. Gray lines denote standard deviation of monthly posts.

**Figure 2:**
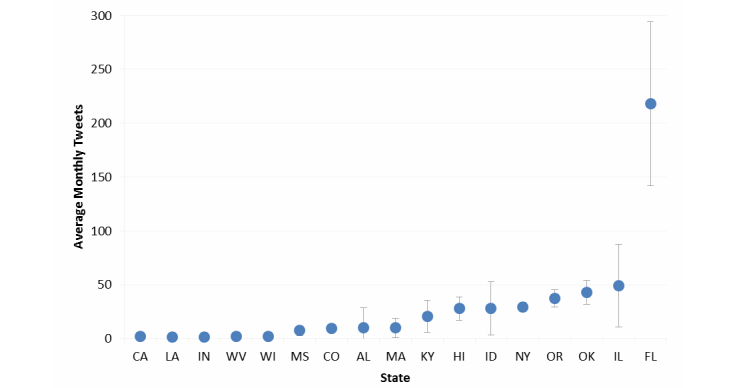
Average number of monthly tweets by state tobacco control programs (n=17), January to December 2013. Gray lines denote standard deviation of monthly tweets. Data for AZ are not included in the graph because there was no posted content to its Twitter site in 2013.

Activity was examined on the 3 most popular social media sites: Facebook, Twitter, and YouTube. Figures 1 and 2 show the average monthly frequency with which TCPs posted content to their Facebook site and disseminated content via Twitter from January to December 2013.

Of the 30 states with Facebook pages, most (22/30, 73%) posted content (ie, messages, photos, videos) an average of 6 to 9 times per month. Two states, Florida and Illinois, posted content 10 times or more per month on average (Florida: mean 61, SD 12; Illinois: mean 14, SD 9). The median number of yearly Facebook posts was 89 (range 0-727). Among all TCP posts coded for content from March to June 2013, 39.70% (705/1776) included photos and 7.66% (136/1776) included videos.

Fewer state TCPs used Twitter than Facebook. However, those that used Twitter delivered more content through Twitter than Facebook. On average, Florida posted content most often at 220 times per month and 6 other states posted at least once per day. The median number of yearly TCP tweets was 118 (range 15-2618). Among all TCP tweets coded, 10.69% (120/1123) included links to photos and 6.06% (68/1123) included links to videos.

In 2013, the median number of videos posts to TCP YouTube sites was 39 (range 3-276). Videos consisted primarily of uploaded antitobacco television advertisements.

### Audience Reach

The reach of social media sites varied widely by platform and by state in 2013 (Table 1). Overall, the level of exposure to social media sites was low relative to states’ populations. The median number of people who liked a TCP Facebook site was 893; however, the range was 72 to 226,722, suggesting dramatic variation in reach. Among TCPs with a Facebook page, 73% (22/30) had less than 100 likes per 100,000 adults in the state, and 13% (4/30) had more than 400 likes per 100,000 adults. Overall, the median number of Twitter followers was lower than Facebook likes at 122 (range 13-2690). Among TCPs with a Twitter page, 61% (11/18) had less than 10 followers per 100,000 adults and only 1 state (Hawaii) had more than 100 followers per 100,000 adults. The median number of views of TCP videos on YouTube was 5216 (range 34-1,308,248); however, there was enormous variation across TCPs. For example, the yearly video views of Florida’s YouTube channel alone accounted for 60% of all video views for the 20 state TCP YouTube sites.

### Audience Engagement

There was considerable variation in the level of audience engagement on TCP social media sites from March to June 2013 (Table 2). The 4 TCPs that generated the most audience engagement through Facebook were California, with a median of 114 (range 5-1370) engagement activities per post (ie, through post likes, comments, or shares), Florida (median 116, range 5-1277), South Dakota (median 53, range 10-461), and Alaska (median 33, range 0-261). In addition to these 4 states, posts in a few other states had high levels of audience interaction. For example, 81% (25/31) of Minnesota’s TCP Facebook posts were commented on. Similarly, Kentucky, New York, Oklahoma, and Utah had at least 1 like on 90% or more of their posts. Across all states, the median of 27% (range 0-95) of Facebook posts had at least 1 comment.

Compared with Facebook users, Twitter users were less likely to share content with their social circle. On average, nearly 1 in 10 TCP tweets (9.68%, 537/5547) were retweeted during the study period. In 2013, Florida’s Twitter content was shared with others most frequently (40.56%, 1062 of 2618 TCP tweets retweeted), followed by Oklahoma (23.1%, 119/515). Another 7 states had between 20 and 65 TCP tweets shared with others in 2013 (Hawaii, Idaho, Illinois, Kentucky, Massachusetts, New York, and Oregon), and the remaining 8 states on Twitter had fewer than 10 TCP retweets (Figure 2). Across TCP YouTube sites, less than one-third of all posted videos (30.5%, 180/591) received at least 1 like. Also, 1 in 6 videos (15.6%, 92/591) received 1 comment or more from viewers.

### Promotion of State Cessation Services and the Tips Campaign

During the airing of the *Tips* campaign from March to June 2013, 64% (16/25) of TCP Facebook sites had 5 or fewer posts that promoted cessation services. Across all sites, approximately 15% (14.60%, 164/1123) of all TCP Facebook posts included materials such as television advertisements or links to CDC’s *Tips* campaign. In total, 64.11% (720/1123) of all TCP Facebook posts coded included no promotion of either telephone quitlines or Web-based cessation services available to smokers.

Of the TCP Twitter sites in use over the 4-month period of *Tips*, 56% (9/16) had 6 or fewer tweets that promoted cessation services. In total, 17.85% (317/1776) of all Twitter content included materials such as television advertisements or links to CDC’s *Tips* campaign while it was on air. On average across states, 75.00% (1332/1776) of all TCP Twitter content included no promotion of either telephone quitlines or Web-based cessation services available to smokers.

**Table 1:** Date of site establishment and reach metrics for TCP social media sites (N=30).

State	Facebook			Twitter			YouTube		
	Page start date	Number of page likes	Likes per 100,000 adults	Date of first tweet	Followers	Followers per 100,000 adults	Channel start date	Total views	Total videos
AL	8/20/09	411	11.2	4/23/10	77	2.1	—	—	—
AK	6/14/10	2257	422.4	—	—	—	—	—	—
AZ	6/4/10^a,b^	512	10.5	6/27/12	13	0.3	4/26/12	4170	58
AR	2/16/10	848	38.1	—	—	—	1/24/08	43,287	277
CA	12/1/09	59,309	208.7	3/8/13	18	0.1	12/9/09	470,835	28
CO	6/21/11	787	20.2	6/11/12	96	2.5	4/11/13	616	14
CT	7/26/11	2328	83.8	—	—	—	7/26/11	34	6
FL	1/28/08	226,722	1505.1	3/29/10	2690	17.9	1/28/08	1,308,248	60
HI	12/30/10	4686	437.9	7/28/09	1688	157.7	7/6/09	6262	36
ID	5/14/09	1480	127.9	9/1/09	519	44.9	—	—	—
IL	10/29/13	1313	13.4	10/29/13	59	0.6	—	—	—
IN	12/9/09	1672	34.0	7/28/11	170	3.5	12/9/09	1215	4
KY	9/16/11	526	15.7	9/20/11	100	3.0	12/21/11	1199	3
LA	1/14/11	547	15.8	2/14/11	377	10.9	1/13/12	65	1
MA	4/8/09	937	18.1	1/26/11	620	12.0	3/5/09	14,525	62
MN	8/5/09	2421	59.5	—	—	—	11/1/09	44,993	41
MS	7/7/11	130	5.8	7/8/11	80	3.6	5/12/11	686	23
NE	5/16/11	1086	78.5	—	—	—	—	—	—
NV	10/26/10	83	4.0	—	—	—	—	—	—
NH	5/23/11	408	39.3	—	—	—	7/26/10	1243	13
NY	7/15/09	805	5.3	11/25/09	143	0.9	6/18/10	20,863	17
ND	8/27/13	72	13.5	—	—	—	1/23/12	2118	3
OK	11/22/11	2658	93.1	11/12/12	329	11.5	11/21/13	146	1
OR	12/31/09	8416	279.8	1/4/10	1369	45.5	9/21/11	80,578	8
RI	12/12/12^a^	88	10.6	—	—	—	—	—	—
SD	1/10/11	9344	1504.8	—	—	—	—	—	—
UT	3/23/11	740	38.2	—	—	—	5/31/11	34,557	81
VT	11/2/09	1417	283.2	—	—	—	12/28/12	140,324	19
WV	3/16/10	611	41.5	3/16/10	37	2.5	—	—	—
WI	4/26/10	159	3.6	8/13/09	70	1.6	—	—	—
Median	6/14/10	893	38	9/9/10	122	3	5/21/11	5216	18
Min	1/28/08	72	4	7/28/09	13	0	1/24/08	34	1
Max	10/29/13	226,722	1505	10/29/13	2690	158	11/21/13	1,308,248	277

^a^ No start date recorded; date of first post.

^b^ Last post August 23, 2012.

**Table 2:** Audience engagement with TCP Facebook sites and content of posts, March to June 2013.

State^a^	TCP posts, n	Audience interaction and engagement				Promotional posts, n (%)		Post features, n (%)	
		Audience activity^b^ per post, median (range)	Posts, n (%)			*Tips* campaign	State cessation services^c^	Posts with video	Posts with photo
			With likes	With shares	With comments				
AL	46	1 (0-9)	15 (33)	11 (24)	5 (11)	8 (17)	10 (22)	4 (9)	24 (52)
AK	58	33 (0-261)	56 (97)	36 (62)	40 (69)	2 (3)	32 (55)	0 (0)	52 (90)
AR	12	2 (0-10)	7 (58)	2 (17)	4 (33)	2 (17)	2 (17)	0 (0)	1 (8)
CA	48	114 (5-1370)	48 (100)	45 (94)	45 (94)	6 (13)	10 (21)	3 (6)	28 (58)
CO	44	3 (0-87)	31 (71)	12 (27)	6 (14)	11 (25)	13 (30)	5 (11)	24 (55)
CT	48	3 (0-40)	37 (77)	11 (23)	16 (33)	0 (0)	1 (2)	0 (0)	2 (4)
FL	231	116 (5-1277)	231 (100)	229 (99)	219 (95)	8 (4)	94 (41)	13 (6)	134 (58)
HI	28	2 (0-7)	20 (71)	1 (4)	5 (18)	1 (4)	6 (21)	0 (0)	14 (50)
ID	44	1 (0-14)	27 (61)	6 (14)	3 (7)	0 (0)	3 (7)	2 (5)	8 (18)
IN	60	4 (0-23)	48 (80)	48 (80)	16 (27)	16 (27)	38 (63)	11 (18)	39 (65)
KY	38	7 (0-27)	35 (92)	11 (29)	8 (21)	1 (3)	0 (0)	3 (8)	16 (42)
LA	17	2 (0-15)	13 (77)	7 (41)	3 (18)	9 (53)	5 (29)	2 (12)	7 (41)
MA	35	4 (0-11)	27 (77)	15 (43)	10 (29)	2 (6)	2 (6)	1 (3)	25 (71)
MN	31	10 (0-107)	30 (97)	16 (52)	25 (81)	0 (0)	17 (53)	6 (19)	3 (10)
MS	41	1 (0-5)	15 (37)	10 (24)	2 (5)	30 (73)	2 (5)	5 (12)	28 (68)
NE	42	4 (0-102)	35 (83)	8 (19)	16 (38)	2 (5)	17 (41)	1 (2)	23 (55)
NY	38	5 (0-11)	36 (95)	14 (37)	9 (24)	0 (0)	19 (50)	1 (3)	3 (8)
OK	47	14 (1-120)	47 (100)	21 (45)	29 (62)	5 (11)	31 (66)	6 (13)	18 (38)
OR	50	6 (0-41)	40 (80)	17 (34)	24 (48)	8 (16)	20 (40)	2 (4)	17 (34)
RI	50	2 (0-9)	30 (60)	14 (28)	3 (6)	6 (12)	13 (26)	4 (8)	8 (16)
SD	31	53 (10-461)	31 (100)	28 (90)	25 (81)	5 (16)	18 (58)	2 (7)	25 (81)
UT	54	12 (4-215)	54 (100)	51 (94)	15 (28)	0 (0)	43 (80)	44 (82)	1 (2)
VT	16	1 (0-14)	6 (38)	7 (44)	2 (13)	3 (19)	3 (19)	3 (19)	4 (25)
WV	7	2 (0-17)	5 (71)	3 (43)	0 (0)	1 (14)	2 (29)	1 (14)	3 (43)
WI	7	1 (0-3)	4 (57)	2 (29)	0 (0)	2 (29)	2 (29)	3 (43)	0 (0)
Median	42	4 (0-1370)	31 (77)	12 (37)	9 (27)	2 (12)	10 (29)	3 (8)	16 (42)

^a^ Five states (Arizona, Illinois, Nevada, New Hampshire, and North Dakota) with Facebook sites did not post content during the time period.

^b^ Number of individual post likes, comments, or shares.

^c^ Post contains the state or national telephone quitline number and/or Web-based cessation services.

## Discussion

Although 30 state TCPs adopted at least 1 form of social media, only 7 disseminated social media messages on a daily basis in 2013. Most content on TCP social media sites did not promote cessation services. Moreover, few state TCPs take advantage of the social media features that generate the most audience engagement—sharing photos and videos—or the opportunity to link to related content, such as the CDC’s *Tips* campaign. Perhaps as a result of these social media practices, most TCPs do not generate a substantial amount of audience engagement, limiting the value of potentially dynamic, interactive communication resources in tobacco control.

Our results corroborate other research indicating that the application of social media to public health research and practice has not taken advantage of its potential for multidirectional communication [27,28]. Study findings suggest that most state TCPs could benefit from strategic communication plans for social media to increase reach and encourage audience interaction and engagement [16]. The Florida TCP, the most prolific user of social media in 2013, presents an example of how effective use of social media can generate audience engagement. During the study period, Florida’s TCP promoted an average of 11 daily posts to its sites, and followers of the Florida TCP retweeted 41% of all content posted on Twitter. On average, there were more than 185 interactions for each posting to Florida’s Facebook site (ie, people who liked, commented on, or shared content), expanding the reach of each individual message. The most successful TCP social media sites communicated information in a way that reflected the audience preferences (eg, with videos and photos), thereby encouraging audience response and discussion. Without engaging in interactive communication with their audience, state TCPs are unable to characterize their audience or receive feedback for site improvements [28].

Taken together, these findings indicate that changes in the social media practices of state TCPs can enhance their ability to connect with constituents—including smokers who are interested in quitting—in ways that may promote cessation. State TCPs have a long history of providing evidence-based cessation services. Although improvements to the content and interactive nature of TCP social media sites are contingent upon their ability to dedicate human resources to site management, states have access to well-designed tobacco cessation online materials from a variety of government and nonprofit agencies (eg, CDC’s Office on Smoking and Health, Public Health Service, National Cancer Institute). To encourage participation, TCPs should focus on the dissemination of evidence-based tobacco cessation information in an engaging format promote appropriate behavioral interventions, and create an online environment that increases participation and 2-way communication with users.

Active TCP social media sites could influence how smokers discover, access, and understand cessation- and tobacco-related information, including connecting the public to telephone quitlines and Web-based cessation services. Traditional media campaigns are often most effective when accompanied by access to available services [29]. Social media messages can reach consumers with both information and links to services, such as telephone quitlines. In addition, social media sites present a unique opportunity to address state TCP goals for increasing cessation because they have a very low barrier for affıliation and present smokers with the option to join large ad hoc networks within their state [30]. TCP-driven content and interactions with users should be aimed at increasing smokers’ use of evidence-based treatment options and enhancing self-efficacy during the quitting process.

As private and government organizations move toward online technologies to promote and provide services, there is a greater need for public health practitioners to be able to create evidence-based websites and promote strategies that will maximize exposure to evidence-based cessation services [31]. State TCPs face a wide range of barriers, including lack of dedicated staff within state health departments to encourage improvements to social media, lack of knowledge regarding best practices in social media, lengthy approval processes or policy restrictions that hinder improvements, and financial limitations for cessation-related promotional activities and services [3,29]. Large-scale and rapid improvements to state TCP social media sites may be bolstered by active collaboration among state and federal agencies, both of whom seek to improve population health outcomes by reducing smoking prevalence.

Although the emergence of social media sites presents a unique opportunity for state TCPs to communicate and engage with smokers, literature is lacking to evaluate the effectiveness of these channels in promoting cessation services. Some evidence suggests that online interaction and connectedness can increase cessation self-efficacy [32]. Wide-scale adoption of improvements to the content and interactive nature of TCP social media sites may encourage supportive communities of online smokers and yield increases in the use of evidence-based interventions. Social network analysis may help us better understand how federal and state TCPs are connected and how these networks can be leveraged to help inform more strategic communications plan. More experimental and applied research across multidisciplinary teams (eg, social media researchers, federal and state tobacco control cessation managers, and marketers) is necessary to understand the conditions under which TCP social media sites successfully engage and motivate smokers to quit.

This study has several limitations. Data could not be captured for messages that were deleted, noted as private by a TCP, or shared offline. Also, some TCPs may not have promoted state cessation services heavily through social media during the airing of the national media campaign, but may have done so during other time periods. Further, this study provides a quantitative summary of audience interaction and did not examine the type of site users or systematically examine users’ posted content. Although state TCPs primarily target smokers in their campaigns, audiences following state TCPs may also include other health agencies [18]. Similarly, some TCP sites may be linked to cessation programs not intended for smokers (eg, California’s Twitter). Future research on the type of site users and posted content should yield a better understanding of the level of engagement by smokers as well as public health organizations and other audiences [33]. Despite its limitations, this study provides an important first look at state TCP social media sites and audience engagement.

## Multimedia Appendix 1

State TCP Facebook, Twitter, and YouTube Links, December 2013.

## Abbreviations

TCP: tobacco control program
